# Differentiation in putative male sex pheromone components across and within populations of the African butterfly *Bicyclus anynana* as a potential driver of reproductive isolation

**DOI:** 10.1002/ece3.2298

**Published:** 2016-07-29

**Authors:** Paul M. B. Bacquet, Maaike A. de Jong, Oskar Brattström, Hong‐Lei Wang, Freerk Molleman, Stéphanie Heuskin, George Lognay, Christer Löfstedt, Paul M. Brakefield, Alain Vanderpoorten, Caroline M. Nieberding

**Affiliations:** ^1^ Evolutionary Ecology and Genetics GroupBiodiversity Research CentreEarth and Life InstituteUniversité catholique de LouvainCroix du Sud 4‐51348Louvain‐la‐NeuveBelgium; ^2^ Biological SciencesUniversity of BristolWoodland RoadBristolBS8 1UGUK; ^3^ Department of ZoologyUniversity Museum of ZoologyUniversity of CambridgeDowning StreetCambridgeCB2 3EJUK; ^4^ Department of BiologyPheromone GroupLund UniversitySE‐223 62LundSweden; ^5^ Indian Institute of Science Education and Research ThiruvananthapuramVanasiri Evolutionary Ecology LabCollege of Engineering Trivandrum CampusTrivandrum695016KeralaIndia; ^6^ Laboratory of Analytical ChemistryDepartment of AgroBioChemGembloux Agro‐Bio TechUniversity of LiegePassage des Déportés 2B‐5030GemblouxBelgium; ^7^ Biologie de l’évolution et de la conservationUniversity of LiègeB22 Sart TilmanB‐4000LiègeBelgium

**Keywords:** Lepidoptera, male sex pheromone, mitochondrial introgression, population divergence, reproductive isolation, speciation

## Abstract

Sexual traits are often the most divergent characters among closely related species, suggesting an important role of sexual traits in speciation. However, to prove this, we need to show that sexual trait differences accumulate before or during the speciation process, rather than being a consequence of it. Here, we contrast patterns of divergence among putative male sex pheromone (pMSP) composition and the genetic structure inferred from variation in the mitochondrial cytochrome oxidase 1 and nuclear CAD loci in the African butterfly *Bicyclus anynana* (Butler, 1879) to determine whether the evolution of “pheromonal dialects” occurs before or after the differentiation process. We observed differences in abundance of some shared pMSP components as well as differences in the composition of the pMSP among *B. anynana* populations. In addition, *B. anynana* individuals from Kenya displayed differences in the pMSP composition within a single population that appeared not associated with genetic differences. These differences in pMSP composition both between and within *B. anynana* populations were as large as those found between different *Bicyclus* species. Our results suggest that “pheromonal dialects” evolved within and among populations of *B. anynana* and may therefore act as precursors of an ongoing speciation process.

## Introduction

As Darwin (1871) already noticed and recent studies have repeatedly demonstrated, sexual traits are often the most divergent characters among closely related species (Mendelson and Shaw 2005; Arnegard et al. 2010; Safran et al. 2012). Large differences in sexual traits have therefore been postulated as the signature of ongoing evolution of reproductive isolation and recent speciation (Allender et al. 2003; Boul et al. 2007; Ritchie 2007). In this scenario, divergence in sexual traits within a species contributes to reproductive isolation and thus speciation. However, interspecific differences in traits might accumulate after speciation and be the consequence rather than the cause of speciation (Butlin 1987). Hence, a potential link between trait divergence and speciation can only be made if we observe trait differences that have evolved before or during the speciation process, that is, at the level of populations or early stages of differentiation (Tregenza et al. 2000; Masta and Maddison 2002; Ritchie 2007; Amézquita et al. 2009; Grace and Shaw 2012; Oneal and Knowles 2013; Schwander et al. 2013).

Among the sexual traits potentially involved in differentiation, sex pheromones, which convey information related to mate choice among conspecific individuals in many species of mammals, reptiles, amphibians, fishes, and insects (see Kikuyama et al. 2002; Johansson and Jones 2007; Smadja and Butlin 2009; Wyatt 2014 for review), may play a key role. Indeed, species specificity of sex pheromones maintains reproductive isolation between species sharing the same habitat (Löfstedt et al. 1991; Roelofs et al. 2002). Furthermore, there is evidence for intraspecific quantitative and qualitative variation in sex pheromones in Lepidoptera that may form a basis for intraspecific mate choice (e.g., *Agrotis segetum*: Toth et al. 1992; LaForest et al. 1997; *Ctenopseustis obliquana*: Clearwater et al. 1991; *Ostrinia nubilalis*: Klun et al. 1975; Roelofs et al. 1985). Among the taxa with intraspecific chemical divergence, some also display a strong genetic differentiation, which questions whether these populations are still genetically compatible, that is, populations of the same species (White and Lambert 1995; Sperling et al. 1996; Svensson et al. 2013). Therefore, we suggest that it may be helpful to compare pheromone differentiation and genetic differentiation in order to assess whether chemical signal may play a role in speciation.

The sub‐Saharan butterfly genus *Bicyclus* (Lepidoptera, Satyrinae) is a suitable model to explore the role of pheromone divergence in the process of speciation. It includes close to 100 species, and up to 20 species can be found in sympatry in a single forest patch (Condamin 1973; Aduse‐Poku et al. 2012; Valtonen et al. 2013). In this genus, males have wing patches and brushes formed by modified scales that are thought to produce and emit the male sex pheromones (MSP). The importance of chemical sexual traits in the differentiation among species was suggested by the fact that differences in the position and shape of the androconia are used for species identification in the field (Condamin 1973). Recently, we identified the major male‐specific compounds (putative MSP, hereafter pMSP) in 32 field‐caught *Bicyclus* species and unraveled a pattern of reproductive character displacement at the scale of the genus (Bacquet et al. 2015). Between the closely related species of the genus, we observed on average five differences in the presence of pMSP components (Bacquet et al. 2015). This supports the role of theses major male compounds in species recognition and potentially in *Bicyclus* speciation (Bacquet et al. 2015). Hence, we tested whether divergence in MSP composition accumulates before completion of genetic isolation within a species, using *Bicyclus anynana* (Butler, 1879) as a model. In this species, three MSP components have been identified using physiological (gas chromatography coupled to electroantennography; GC‐EAD hereafter) and behavioral mate choice experiments (Nieberding et al. 2008). These three MSP components, (*Z*)‐9‐tetradecen‐1‐ol (hereafter MSP1), hexadecanal (MSP2), and 3: 6,10,14‐trimethylpentadecan‐2‐ol (MSP3), are used at short range when males flicker their wings and erect their androconial hair probably favoring their dissemination (Nieberding et al. 2008). Variation in the abundance of the three MSP components among males indicates age and level of inbreeding of the males and is under sexual selection by females (Nieberding et al. 2008, 2012; San Martin et al. 2011; van Bergen et al. 2013). If the divergence of male chemical profiles is involved in speciation, we expect to observe MSP differences between, or even within, populations of the same species, that is, before completion of the speciation process.

## Material and Methods

### Population sampling

**Figure 1:**
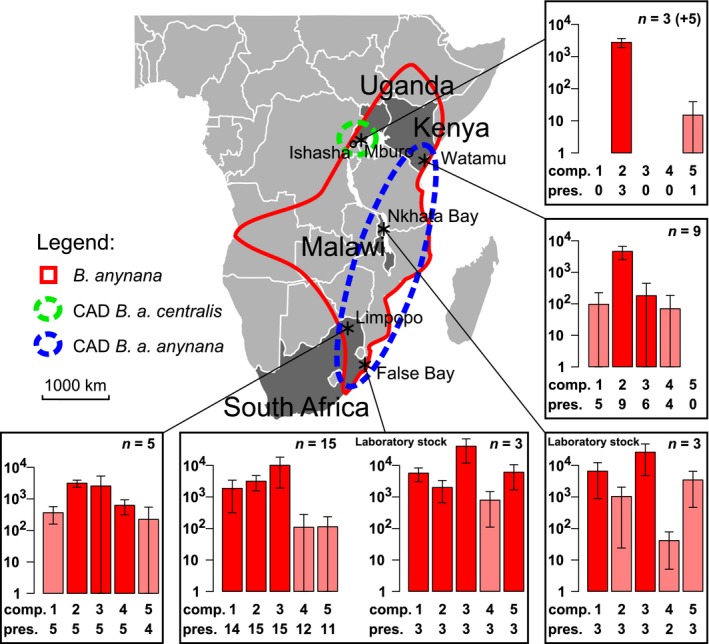
Location and putative male sex pheromone (pMSP) composition of the *Bicyclus anynana* populations. Sampling sites are represented by asterisks excepted for Ishasha where only genetic data were sampled. Hypothetical repartition range derived from Condamin (1973) is represented in plain red. The two CAD haplotypes corresponding to *B. anynana* subspecies are in green and blue dashed lines. The bar plots represent the log‐transformed average amount (ng) of each pMSP component in each population (±SD). The sample size is given for each graph (within brackets for the preliminary samples from Uganda). The number below each bar (“comp.”) codes the pMSP component: 1: (*Z*)‐9‐tetradecen‐1‐ol; 2: hexadecanal; 3: 6,10,14‐trimethylpentadecan‐2‐ol (the three active MSP identified from the laboratory stock of *B. anynana*, Nieberding et al., 2008); 4: unidentified anynana #19; 5: octadecan‐1‐ol (physiologically active in GC‐EAD tests of individuals from False Bay, Nieberding et al., unpublished data); (see Table S2 and Fig. S1 for details of their identification). The abundance of each pMSP component selected once is represented for all the populations. The counts (“pres.”) below the compounds number represent the number of individuals displaying the pMSP component in the population. The bars are light colored for the populations in which the compound was not selected as a pMSP component.

We sampled four geographically distinct wild populations of *B. anynana* (Fig. 1 and Table 1). Notably, the populations in Uganda are of the subspecies *Bicyclus anynana centralis* Condamin, 1968, while others are regarded *Bicyclus anynana anynana* Condamin, 1968. Among the 160 sampled individuals, a subset of 35 males and 33 females was used for the chemical analyses (Table 1). Most of the males and females in each location were sampled during a single trapping session of 1 week (Tables 1 and S1). We also sampled *B. anynana* individuals from two laboratory stocks, the first one originating from Malawi (Nkhata Bay, from 80 females sampled in 1988, Brakefield et al. 2009) and used in most studies on *B. anynana*, and the second one sampled in 2006 from South Africa (False Bay, from over 70 females, sampled in 2006, de Jong et al. 2010).

**Table 1:** Location and size of the samples for genetic and chemical analyses of *Bicyclus anynana*. Details for each individual are given in Table S1

Country	Location	Number of sequences			Number of chemical analyses		Date of chemical sampling
		COI	CAD	EF1*α*	♂	♀	
Uganda	Mburo	38	12	7	3	2	9/2009
Uganda	Ishasha	11	11				
Kenya	Watamu	27	16		9	8	3/2007
Malawi laboratory stock	Nkhata Bay	9			3	2	6/2010
South Africa	False Bay	37	28	2	15	15	♀ 7/2006, ♂ 5/2007
South Africa laboratory stock	False Bay	8			3	2	5/2010
South Africa	Limpopo	30	10	3	5	6	7/2006
	Total	160	77	12	38	35	

### DNA sequencing

DNA was extracted with the DNeasy Blood and Tissue Kit (Qiagen) from a leg or a thorax piece kept in 100% ethanol at −20°C or in glassine envelope at −80°C. In total, we sequenced 160 and 77 individuals, respectively, for the mitochondrial gene cytochrome oxidase 1 (COI) and the nuclear gene carbamoyl‐phosphate synthetase 2, aspartate transcarbamylase, and dihydroorotase (CAD) following the protocols of Monteiro and Pierce (2001) and Wahlberg and Wheat (2008, see Table 1 and www.nymphalidae.net/Nymphalidae/Molecular.htm). Some COI sequences were already available from de Jong et al. (2011). A second nuclear marker, elongation factor alpha 1 (EF1*α*), was sequenced for a subset of 12 individuals. The alignments were 853, 714, and 1243 base pairs long and presented 34, 51, and 18 SNPs for COI, CAD, and EF1*α*, respectively. Sequences are stored in GenBank (accession numbers in Table S1).

### Analysis of population genetic structure and diversity

We deduced the haplotypes forming each individual CAD diploid genotypes using the Excoffier–Laval–Balding algorithm (ELB) in Arlequin software (version 3.5.1.2) with default parameter values (Excoffier et al. 2003, 2005). Five independent runs of the ELB algorithm were compared, and we discarded individuals showing different coding phases across runs (two individuals, Table S1).

To represent the genetic distance among haplotypes, we constructed median‐joining networks with the software Network 4.6.1.1 (Bandelt et al. 1999, www.fluxus-engineering.com). To ensure the consistency of the network construction, several reconstructions were performed with variable epsilon values, and the less parsimonious links were deleted to simplify the network (Polzin and Daneshmand 2003). We also tested the consistency of network links by analyzing partial datasets after removing the haplotypes occupying a central position in each full network. For the sake of comparison, we additionally reconstructed the maximum‐likelihood phylogenetic trees (Appendix 2).

We investigated the distribution of the genetic diversity among populations with an analysis of molecular variance (AMOVA) based on haplotype uncorrected pairwise differences (Excoffier et al. 1992). We performed 1000 permutations of the individuals among populations to test the significance of the observed pattern of genetic variation. We also calculated Φ_ST_ between pairs of populations, a type of *F*_ST_ based on haplotype dissimilarity (Excoffier et al. 1992). We run these analyses in Arlequin (version 3.5, Excoffier et al. 2005).

### Extraction, analysis, and repeatability of chemical profiles

Androconia were dissected from the wings of one side of freshly killed butterflies or from butterflies frozen alive and kept at −80°C. At −80°C, the chemical compounds do not evaporate enough to induce differences of extraction profiles between these two treatments (P. Bacquet, unpublished data). Each androconium was extracted in separate 2‐mL screw cap vials (VWR International, Leuven, Belgium) with 100 μL of redistilled *n*‐heptane (VWR International) containing 1 ng/μL of (*Z*)‐8‐tridecenyl acetate (Z8‐13:OAc) as an internal standard. The remaining tissue of each dissected wing was also extracted in a vial with 300 μL redistilled heptane containing 0.33 ng/μL of the internal standard. The wing extracts were analyzed on Agilent (Santa Clara, CA) 5975 mass‐selective detector coupled to an Agilent 6890 gas chromatograph (GC‐MS), equipped with a HP‐5MS capillary column (30 m × 0.25 mm i.d., and 0.25 μm film thickness; J&W Scientific, Folsom, CA). The oven temperature was programmed from 80°C for 3 min, then to 210°C at 10°C/min, hold for 12 min and finally to 270°C at 10°C/min, hold for 5 min. Inlet and transfer line temperatures were 250 and 280°C, respectively, and helium was used as the carrier gas with a flow of 0.8 mL/min. Compounds that eluted no later than *n*‐tricosane were considered as the more volatile part of the chemical diversity displayed by these butterflies, and thus included in our analysis. Identification of wing compounds was performed by comparing the GC retention times and mass spectra with commercially acquired or laboratory‐synthesized authentic compounds on both nonpolar (HP‐5MS) and polar (INNOWax, 30 m × 0.25 mm i.d., and 0.25 μm film thickness; J&W Scientific) columns. Tentative structures were assigned for compounds that were not fully identified. The chemical composition and related information are summarized in the Table S2 and Figure S1.

We determined the repeatability of full chemical profiles, that is, of all compounds found in the chemical extracts (as opposed to the pMSP composition, see below), among field‐sampled individuals within and between the four populations. For this purpose, we used Spearman correlations on the amounts of compounds displaying identical mass spectra and retention times across individuals, thereby assumed to be homologous.

### Selection of pMSP components from chemical profiles

The chemical profile of *Bicyclus* butterflies consists of many compounds (Table S2). For example, in the model species *B. anynana*, over a hundred chemicals with lower volatility than fatty acids have been identified on the different body parts, of which twenty significantly correlated with traits important in mate choice in this species (sex and age; Heuskin et al. 2014). As behavioral or GC‐EAD assays would be unrealistic to perform for each field‐caught population, we used a previously developed standardized routine to select a list of pMSP among all the compounds observed per species (Bacquet et al. 2015). The selection criteria were based on physiological (GC‐EAD) and behavioral data accumulated from the stock populations of *B. anynana* originating from Malawi and South Africa (Nieberding et al. 2008, 2012; van Bergen et al. 2013 and unpublished data). In summary, we first applied a threshold of 10% of the internal standard such that any compound at a lower amount than 20 ng per individual was discarded from the analysis to standardize the quality of peak identification among samples. After this filtering step, the absolute amount of each compound and its repeatability level among individuals were computed. A compound was considered repeatable if present in two males out of three. As the three behaviorally and electrophysiologically active MSP components identified in the Malawi stock of *B. anynana* are among the top third most abundant of the repeatable, and male‐specific compounds (Nieberding et al. 2008, 2012; van Bergen et al. 2013), we thus selected in every population the pMSP components following the same criteria. The female samples were used to assess the male specificity of the chemical compounds. A compound was considered as male specific if it was at least five times more abundant, than in female extracts sampled in the same population. In case a compound was selected as a pMSP in one population, its presence in other populations was also included in the analyses of the pMSP composition. This ensured not to artificially exaggerating the difference between a population where a compound is selected as a pMSP component and a population where it is not. Of note, our selection method of abundant and repeatable male‐specific compounds that form the pMSP of *Bicyclus* populations is in accordance with observations that MSP components that experience directional sexual selection are usually more abundant than other compounds (see Steiger et al. 2011; and examples in Lepidoptera: Iyengar et al. 2001; Andersson et al. 2007; Hymenoptera: Ruther et al. 2009; lizards: Martín and López 2010; or elephants: Greenwood et al. 2005).

### Analysis of chemical diversity

We applied three types of dissimilarity indices on the chemical profiles of individuals. First, the Jaccard dissimilarity described qualitative differences in composition (presence and absence of compounds). Second, the Euclidean distance discriminated chemical profiles based on the absolute amounts of compounds and, thirdly, on the relative amounts of compounds (proportion of the sum of all compounds per individual). Using these dissimilarity indices, we performed permutational multivariate analyses of variance for estimating how much chemical variation was explained by sex and geographic origin of the individuals (PerMANOVA, adonis function in R package vegan; Oksanen et al. 2011). PerMANOVA was preferred to the conventional MANOVA because its nonparametric permutational approach for testing significance is robust to the presence of large proportions of null data and is not restricted to the use of Euclidean distances (McArdle and Anderson 2001).

### Comparison of genetic and chemical differentiations between pairs of populations

If, as we hypothesized, chemical divergence is a potential cause of reproductive isolation and speciation in *Bicyclus* butterflies, the level of pMSP differentiation should on average be as large as, or larger, than the level of neutral genetic differentiation between corresponding pairs of populations. We thus compared Φ_ST_ between all pairs of populations based on either chemical distances or genetic CAD‐based distances in the similar framework with Arlequin (version 3.5, Excoffier et al. 1992, 2005). For chemical profiles, we used distance matrices based on either presence–absence of compounds or absolute amounts of compounds or relative amounts of compounds.

## Results

### Patterns of genetic structure

**Figure 2:**
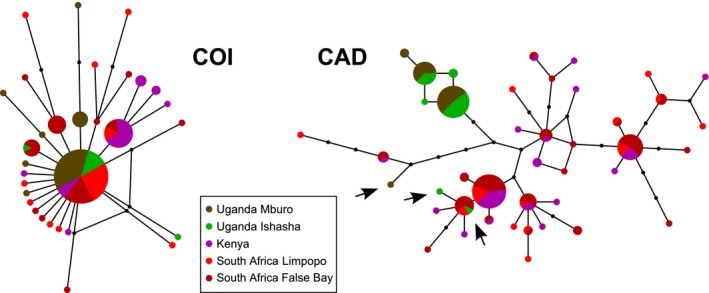
Networks of the COI (left) and CAD (right) genes for *Bicyclus anynana*. The surface of a circle is proportional to the number of individuals bearing one haplotype. Black circles represent intermediate haplotypes missing from the sampling. For the sake of representation of the COI network of *B. anynana*, the distance between haplotypes is twice as large as in the CAD network. We only represented the simpler networks built with a null epsilon value as other reconstruction parameters showed consistent similar results. The three arrows pinpoint the probable introgressed haplotypes forming CAD hybrids in the population from Uganda.

**Figure A2:**
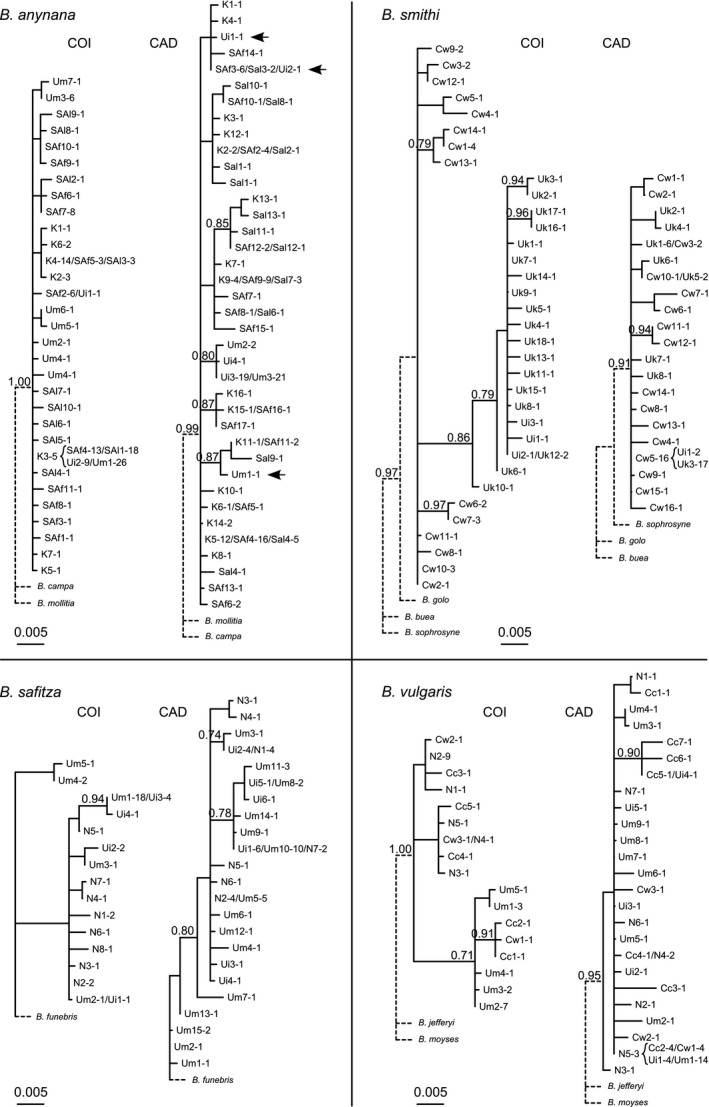
Phylogenetic reconstructions of the species trees using ML on COI and CAD gene sequences. Internal nodes with bootstrap values above 0.50 are represented, and values above 0.70 are specified. Each tip represents a haplotype named according to the geographic origin of sampling (N for Nigeria; Cw and Cc for central and west Cameroon; Uk, Um, and Ui for Kibale, Mburo, and Ishasha in Uganda; K for Kenya; SAf and SAl for False Bay and Limpopo in South Africa; see Fig. A1 and Table S1). Numbers after the dashes indicate the numbers of repeated haplotypes in each population. To save space, the dashed branches linking the species to their outgroups are not scaled. The three arrows pinpoint the CAD haplotypes likely introgressed in the Ugandan population of *Bicyclus anynana*.

The mitochondrial COI gene showed a strong similarity among all individuals. This is not expected for a rapidly evolving marker, in particular when compared to the nuclear CAD which displayed a larger diversity (Figs. 2 and A2). A few sequences for a third independent marker (EF1α, nuclear) confirmed that the uniformity of the COI sequences was not reflecting the population's neutral divergence (Table A3). As a confirmation, neutrality tests *H* of Fay and Wu (2000) and *D* of Tajima (1989) were both significant for the COI but not for the CAD gene (Table A4). Altogether, this information suggests that COI underwent a selective sweep and is therefore inappropriate to describe the neutral genetic divergence of the populations in this species, leading us to discard this locus from subsequent analyses (Appendix 3; but see de Jong et al. 2011).

*Bicyclus anynana* displayed significant genetic variation between populations based on the CAD nuclear marker (37% of the total genetic variance, *P *<* *0.001, see AMOVA in Appendix 4 and Table A5). The median‐joining network and the phylogenetic reconstructions of the CAD gene supported the isolation of a Ugandan clade, in which all but three divergent Ugandan haplotypes clustered together (*n *=* *46, Fig. 2; Appendix 2, and see the three black arrows). The three divergent Ugandan haplotypes assembled with South African and Kenyan haplotypes. They very likely belong to hybrids which displayed both Kenyan or South African, and the typical Ugandan haplotypes (Appendix 5). Together with the observation of a selective sweep in the mitochondrial genome across populations, the presence of hybrids shows that there is ongoing gene flow between *B. anynana* populations.

### Patterns of chemical diversity

**Figure 3:**
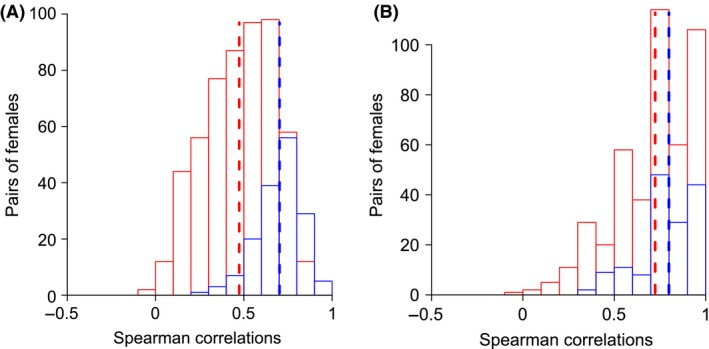
Distribution of Spearman correlations between pairs of *Bicyclus anynana* chemical profiles. For each sex (A: males; B: females), pairs of individuals of the same (in blue) or different (in red) populations were sampled. Dotted lines represent the mean of each distribution.

The total number of chemical compounds per male ranged from 8 to 16 (12.0 ± 3.3; average ± SD) and from one to six in females (2.6 ± 1.2; Appendix 6). Individuals from the same population showed similar chemical profiles (Fig. 3). These profiles were significantly more similar within populations than between (*P *=* *0.001 in males and *P *=* *0.04 in females, test based on 10,000 permutations, Fig. 3).

Sex was the main factor explaining the differentiation of full chemical profiles among individuals (PerMANOVA analysis; *n *=* *73 individuals; significant *R* ^2^ of 47%, 16%, and 67% of the variance in presence, absolute, and relative abundance of the compounds explained by sex, respectively; Table 2). When performing the PerMANOVA analysis on the two sexes separately, the variation in the presence and abundance of compounds was mostly explained by geographic location (PerMANOVA; *n *=* *38 males and *n *=* *35 females; significant *R* ^2^ of 50%, 59%, and 73% for males and of 38%, 38%, and 75% for females; Table A1).

**Table 2:** Distribution of the chemical variation explained by sex and the geographic origin of populations in *Bicyclus anynana*

Sex	Compounds	Factors	Presence–absence (%)	Absolute abundance (%)	Relative abundance (%)
Both	Full profile	Location	**15** ***	**30** ***	**14** ***
		Sex	**47** ***	**16** ***	**67** ***
		Interaction	**8** ***	**20** **	**11** ***
Males	Full profile	Location	**50** ***	**59** ***	**73** ***
	pMSP	Location	**51** ***	**59** ***	**75** ***
Females	Full profile	Location	**38** ***	**38** *	**75** ***

PerMANOVA analyses were performed for each type of chemical distance (presence–absence, absolute, and relative abundances, respectively). In males, the analyses on both full chemical profile and putative male sex pheromone composition are presented. Significant *R* ^2^ are in bold (asterisks inform on significance of the estimates: “*” if *P *≤* *0.05; “**” if *P *≤* *0.01; “***” if *P *≤* *0.001).

We subsequently aimed at spotting the most likely chemical compounds forming the MSP in these populations. One to four pMSP components were selected per population (Fig. 1). We found that the qualitative and quantitative variations in composition of the pMSP in males were mostly explained by geography as for full chemical profiles (PerMANOVA; *n *=* *38; significant *R* ^2^ of 51%, 59%, and 75% for the difference in presence, absolute, and relative abundance of the compounds, respectively; Table 2). Specifically, the three MSP initially identified in the laboratory population of *B. anynana* originating from Malawi were present in all populations of *B. anynana* except in Uganda where MSP1 and MSP3 were absent from the male profile (Fig. 1). Five additional males sampled at the same Ugandan location in 2007 lacked MSP1 and MSP3 as well which suggests it is not an effect due to chance and the small sample size. Of note, these five additional individuals were not added to the analyses because we lacked an internal standard for quantifying the compounds and we did not have the corresponding genetic data. Additional compounds (pMSP4 and pMSP5) were also selected in the South African populations (Limpopo and False Bay laboratory stock, respectively, Fig. 1). The Kenyan population showed some peculiarities regarding the chemical profiles of males. First, some pairs of Kenyan males showed a much poorer correlation (between 0.2 and 0.4) than that found on average between pairs of male chemical profiles (mean = 0.61 ± 0.16 SD, Fig. 3). We also discovered that some Kenyan individuals had the same pMSP profile as the Ugandan population and thus missed MSP1 and MSP3, while other males did not, suggesting that in Kenya, a mixture of different chemical profiles could be present. We found an average number of 2.0 ± 0.0 (mean ± SD) differences in presence and absence of pMSP components between the Ugandan population and the Malawian and South African populations**.** Finally, the pMSPs and the chemical profiles of the two laboratory stocks, and those of the South African laboratory‐adapted and field‐caught populations, were very similar despite over 20 years of laboratory acclimation for the Malawian stock population: All compounds identified in one population were found to be produced at least in some individuals of the other population, demonstrating that the corresponding biosynthetic pathways were conserved between populations (Fig. 1). In summary, qualitative differences in the presence or the absence of population‐specific pMSP components were observed between populations in *B. anynana*, but also within the Kenyan population.

### Comparison of the patterns of genetic and chemical differentiations

**Figure 4:**
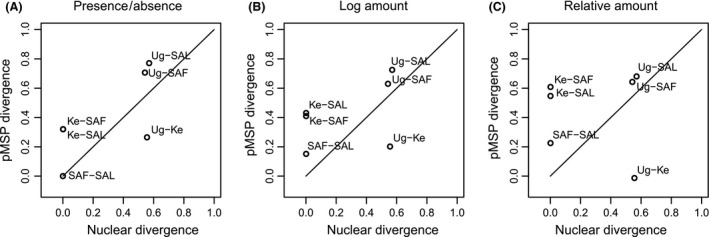
Comparison of the putative male sex pheromone (pMSP) chemical differentiation and of the nuclear genetic differentiation between pairs of *Bicyclus anynana* populations. Graphs are built using Φ_ST_ values for estimating the differentiation of both the pMSP components and of the CAD gene to allow direct comparison. Panels (A‐C) represent the chemical distances based on the presence and absence, the log amounts, and the relative amounts of the pMSP components, respectively. Codes of populations are as follows: Ug, Uganda; SAF and SAL, South Africa False Bay and Limpopo; and Ke, Kenya. A line was placed where genetic and chemical differentiations are identical.

The level of pMSP differentiation between pairs of *B. anynana* populations was higher than the corresponding level of nuclear genetic divergence, and this held true whether the patterns of presence–absence of pMSP components of absolute or of relative variations in abundance in pMSP composition were considered (Fig. 4A–C). Although the number of data points is too limited to test this pattern statistically, it suggests that chemical divergence was concomitant, or preceded, genetic differentiation between *B. anynana* populations. One notable exception to this pattern was the Uganda and Kenya population pair (“Ug–Ke” in Fig. 4), wherein chemical differentiation was lower than neutral genetic divergence. This is because the Kenyan population was composed of genetically similar individuals displaying contrasted chemical profiles with some bearing the Ugandan profile and others the Malawi‐South African one (see Fig. 1 and results above). This increased the average chemical differentiation within the population and decreased the average chemical differentiation between some males from Kenya and the Ugandan population.

## Discussion

We compared variation in pMSPs with neutral genetic variation in an African butterfly in which pMSPs are suspected to play a role in speciation and observed a strong differentiation in both the full chemical profiles and in the pMSP composition among *B. anynana* populations, as well as variation within one population. The chemical differentiation involved both qualitative differences in the composition, and quantitative differences in absolute and relative abundances of chemical compounds. A large part of the variance in chemical profiles could be attributed to the geographic isolation among *B. anynana* populations. We observed particularly obvious differences between the two subspecies of *B. anynana*,* B. a. centralis* in Uganda, and *B. a. anynana* in Malawi and South Africa (Fig. 1; Table 2). Between one and four compounds were selected as pMSP components in each of the *B. anynana* populations (2.0 ± 1.0; mean ± SD) and *B. anynana* subspecies differed by 2.0 **±** 0.0 pMSP components on average. By comparison, we previously selected 3.7 **±** 2.1 pMSP components per *Bicyclus* species, and ~20% of the younger pairs of *Bicyclus* species displayed one to three pMSP differences, while all pairs of *Bicyclus* species differed on average by seven pMSP compounds (Bacquet et al. 2015). Thus, the qualitative differences observed between populations in this study were of the same magnitude as those observed among closely related species and that participate to reproductive isolation across the genus (Bacquet et al. 2015). Moreover, such chemical differences are of greater magnitude than those found in female sex pheromones in moths (Linn and Roelofs 1989). Overall, neutral genetic divergence between pairs of *B. anynana* populations was lower than variation in pMSP although the small sample size for the chemical data could inflate Φ_ST_ values. In addition, a similar level of chemical differentiation was also found within the genetically undifferentiated population of *B. anynana* in Kenya (Figs. 1, 4). Furthermore, there is evidence of ongoing gene flow between all *B. anynana* populations as they share a similar mitochondrial haplotype, and we found three individuals in Uganda that appear to be crosses between the two subspecies. The greater chemical than genetic differentiation lends support to the hypothesis that chemical divergence was concomitant, or preceded, genetic divergence.

This study is one of the few comparing chemical and genetic differentiations at the intraspecific level in animals (White and Lambert 1995; Sperling et al. 1996; Tregenza 2002; Watts et al. 2005; Eltz et al. 2008; Dopman et al. 2010; Palacio Cortés et al. 2010; Runemark et al. 2011; Khannoon et al. 2013; Svensson et al. 2013). For example, Tregenza (2002) and collaborators showed that cuticular hydrocarbon profiles that participate to reproductive isolation vary across European populations of the grasshopper *Chorthippus parallelus*. In several occasions, this genetic approach allowed to unravel that the hypothesized populations were actually cryptic species (White and Lambert 1995; Sperling et al. 1996; Svensson et al. 2013). Our results suggest that variation in sex pheromone composition may be responsible for the evolution of premating isolation among *B. anynana* populations, and hence may act as a driver for speciation.

A follow‐up question would be how frequent is this important divergence of intraspecific chemical profiles in the *Bicyclus* genus? Preliminary data on genetic and chemical distances were gathered for three additional *Bicyclus* species (Appendix 1). In one of the three, *Bicyclus vulgaris*, we could detect qualitative differences of pMSP composition associated to geography similar to what was observed in *B. anynana* (Appendix 6). In addition, *B. vulgaris* displayed genetic differentiation at the mitochondrial level. In contrast, *Bicyclus safitza* and *Bicyclus smithi* did not display clear differences in chemical profiles between sampled populations, although the latter showed mitochondrial differentiation associated to geographic location. Despite the low sample sizes, this suggests that the accumulations of chemical and genetic differences are uncoupled in the *Bicyclus* genus.

Differences in sex pheromone composition among geographically distant populations have been shown to have a genetic basis in several Lepidoptera (Sappington and Taylor 1990a,b; LaForest et al. 1997; Groot et al. 2013). Additional information would be necessary to determine whether the observed chemical differences are genetically fixed or plastic in *B. anynana*. Indeed, we performed chemical analyses on wild individuals sampled at different locations and time periods of the year. While MSP1 and MSP2 ([*Z*]‐9‐tetradecen‐1‐ol and hexadecanal) in *B. anynana* are fatty acid derivatives and synthesized *de novo* (Liénard et al. 2014; Wang et al. 2014), terpenoid compounds such as MSP3 (6,10,14‐trimethylpentadecan‐2‐ol) are likely derived from host plant compounds (Schulz et al. 2011) and potentially affected by host plant use. In addition, climatic or ecological conditions, such as alternative wet and dry seasons typically experienced by these tropical butterflies, could be responsible of part of the observed variation (Landolt and Phillips 1997; Buckley et al. 2003; Groot et al. 2009; Geiselhardt et al. 2012; Larsdotter‐Mellström et al. 2012). The *Bicyclus* genus is known for its seasonal polyphenism with dry and wet season forms varying in life history traits and displaying differences in wing patterns (mostly studied in *B. anynana*; Brakefield and Reitsma 1991; Windig et al. 1994; Brakefield et al. 2009; Prudic et al. 2011). However, several observations argue that part of the observed variation of MSP in *B. anynana* may be genetically based. First, preliminary results in *B. anynana* laboratory population originating from Malawi showed that the MSP composition was neither affected by the temperature during development (the triggering factor of the two seasonal forms) nor by host plant (maize or *Oplismenus sp*., the natural host plant of *B. anynana*; Nieberding et al. 2008). Second, we did not find MSP1 and MSP3 in the population of *B. anynana* from Uganda in five additional males sampled in 2007, which suggests that plasticity was likely not responsible for their particular MSP composition.

To conclude, the pMSP of *B. anynana* displays variations of interspecific magnitude at an intraspecific level (i.e., low genetic differentiation) and is therefore a good candidate to drive reproductive isolation and speciation.

## Conflict of Interest

None declared.

## Supporting information


**Figure S1.** Mass spectra of the pMSP components.

**Table S1.** Detailed list of sampled individuals for the four Bicyclus species.

**Table S2.** List of compounds detected in the four species.

## Figures and Tables

**Figure A1:**
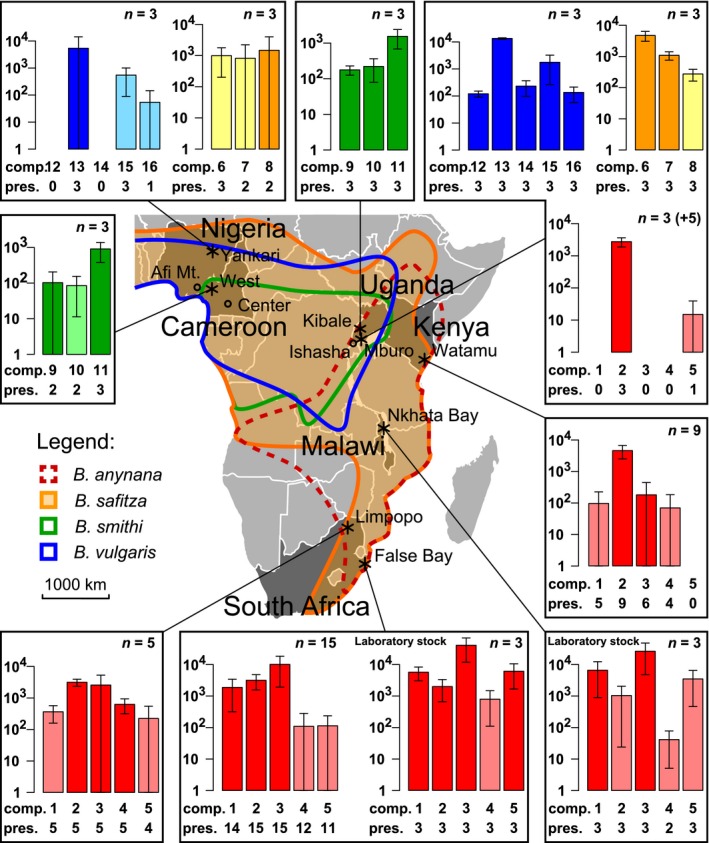
Geographic localization and pMSP composition of *Bicyclus anynana*,* Bicyclus safitza*,* Bicyclus smithi*, and *Bicyclus vulgaris* populations. Sampling sites are represented by asterisks when both DNA and chemical samples were collected, and circles where we only collected DNA samples (Cameroon west: Fossong Ellelem and Bamena; center: Yaoundé, Ebogo and Dja, see Table S1). Hypothetical repartition ranges derived from Condamin (1973) are represented for each species with different colors. The bar plots represent the log‐transformed average amount (ng) of each pMSP component in each population (±SD). The sample size is given for each graph (within brackets for the preliminary samples from Uganda). The number below each bar (“comp.”) codes the pMSP component: MSP1: (*Z*)‐9‐tetradecen‐1‐ol; MSP2: hexadecanal; MSP3: 6,10,14‐trimethylpentadecan‐2‐ol (the three active MSP identified from the laboratory stock of *B. anynana*; Nieberding et al. 2008); pMSP4: unidentified anynana #19; pMSP5: octadecan‐1‐ol (physiologically active in GC‐EAD tests of individuals from False Bay, Nieberding CM, De Jong M, Löfstedt C, unpublished data); pMSP6: 6,10,14‐ trimethylpentadecan‐2‐ol; pMSP7: (*Z*)‐11‐octadecenal; pMSP8: tentatively identified as ethyl (*Z*)‐9‐octadecenoate; pMSP9: (*E*5,*E*9)‐6,10,14‐trimethylpentadeca‐5,9,13‐trien‐2‐yl acetate; pMSP10: octadecyl acetate; pMSP11: tentatively identified as (*Z*)‐9‐tricosene; pMSP12: tentatively identified as 5‐methyl tridecane; pMSP13: 6,10,14‐ trimethylpentadecan‐2‐one; pMSP14: tentatively identified as 3,7,11,16‐tetramethylhexadeca‐2,6,10,14‐tetraen‐1‐ol; pMSP15 and pMSP16: tentatively identified as isomers of 3,7,11,15‐tetramethyl‐2‐hexadecen‐1‐ol (see Appendix 4 for details of their identification). The abundance of each pMSP component selected once in a species is represented for all the populations. The counts (“pres.”) below the compounds number represent the number of individuals displaying the pMSP component in the population. The bars are light colored in the populations where the compound was not selected as a pMSP component.

**Figure A3:**
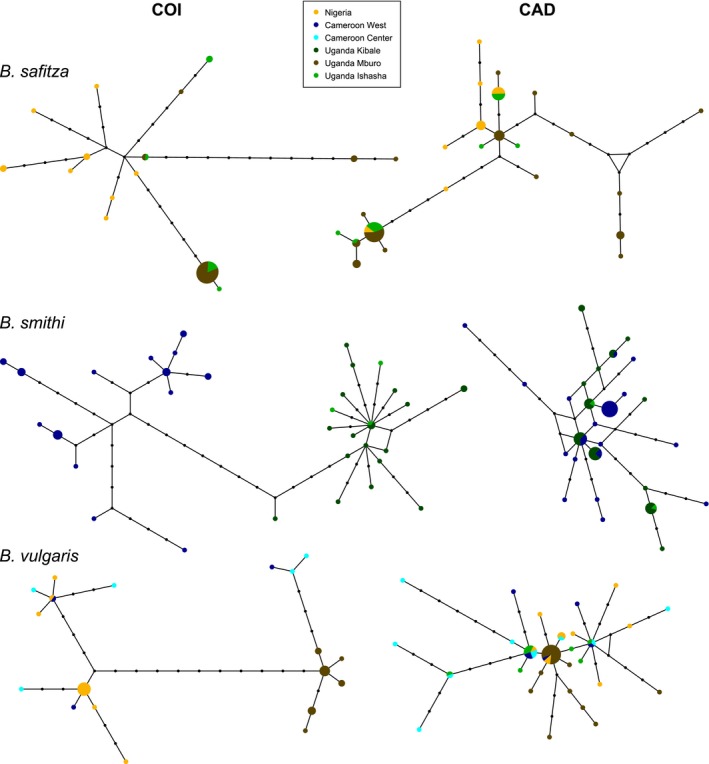
MJ haplotype networks of the two genes for *Bicyclus safitza*,* Bicyclus smithi* and *Bicyclus vulgaris*. The surface of a circle is proportional to the number of individuals bearing this haplotype. Black circles represent intermediate haplotypes missing from the sampling. We only represented the simpler networks built with a null epsilon value (see main text) as other reconstruction parameters showed consistent similar results.

## Appendix 1 Detailed list of sampled individuals for the four *Bicyclus* species

For comparison sake and to investigate the generality of the pattern observed in *Bicyclus anynana*, we analyzed a limited number of samples of three other *Bicyclus* species: *B. safitza* (Westwood, [1850]), *B. smithi* (Aurivillius, 1898), *and B. vulgaris* (Butler, 1868) (*n = *41, 48, and 45, respectively, Table A1). For these, three males and two females per population were used for the chemical analyses (Table A1). The four species can be considered independent at the scale of the genus as they are relatively distant in the phylogenetic tree (10% K_2_P distance on average for the mitochondrial gene COI).

**Table A1:** Summary of the geographical distribution and size of the samples for genetic and chemical analyses of *Bicyclus safitza*,* Bicyclus smithi*, and *Bicyclus vulgaris*. Details for each individual are given in Table S1

Species	Country	Location	Number of sequences		Number of chemical analyses		Date of chemical sampling
			COI	CAD	♂	♀	
*B. safitza*	Nigeria	Yankari	7	7	3	2	10/2008, 2♂ 11/2009
	Nigeria	Afi Mountains	3				
	Uganda	Mburo	23	16	3	2	9/2009
	Uganda	Ishasha	8	8			
*B. smithi*	Cameroon	West	25	16	3	3	4/2009
	Uganda	Kibale	20	15	3	2	10/2009
	Uganda	Ishasha	3	3			
*B. vulgaris*	Nigeria	Yankari	4	4	3	2	10/2008, 1♂ 11/2009
	Nigeria	Afi Mountains	9	1			
	Cameroon	West	3	3			
	Cameroon	Center	5	5			
	Uganda	Mburo	14	11	3	2	9/2009
	Uganda	Ishasha	5	5			
		Total	129	94	18	13	

## Appendix 2 Phylogenetic reconstructions for the four *Bicyclus* species

Maximum‐likelihood (ML) trees were reconstructed using the program Garli (Zwickl 2006; Bazinet and Cummings 2011). The model of sequence evolution GTR + G + I was selected to encompass the features of the best models of all four species and the two genes to allow comparison of phylogenetic reconstructions on a common basis. The model ranking was based on corrected Akaike information criterion computed with JModeltest (Posada 2008). Final consensus trees for each species and each gene were obtained from 2000 bootstrap replicates starting from the best tree of 10 independent ML searches. We rooted the phylogenetic trees with closely related species: *Bicyclus campa* and *B. mollitia* for *B. anynana*;* B. funebris* for *B. safitza*;* B. buea*,* B. golo*, and *B. sophrosyne* for *B. smithi*; and *B. jefferyi* and *B. moyses* for *B. vulgaris* (Condamin 1973; Monteiro and Pierce 2001).

We performed the test of Shimodaira and Hasegawa (1999; S.H. test hereafter) to compare likelihood scores of trees reconstructed with different constraints in order to test more specifically the support for population (defined according to the sampling location) separation in different clades of the trees. We used positive and negative constraints to test the support of population separation in different clades. Positive constraints forced all individuals of the same population to be kept in a single clade in the trees during the reconstruction. Conversely, negative constraints forced the individuals of each population to be distributed across different clades. We used positive constraints to test the support of observed separation of some populations in distinct clades, and negative constraints to test the support of observed admixture of populations. To compare the different hypotheses, each corresponding best tree was imported into R program (Paradis et al. 2004; R Development Core Team 2011); the branch lengths were optimized with the functions pml and optim.pml for the tree likelihood to be subsequently compared with S.H. tests (package Phangorn, Schliep 2011). The likelihoods of the trees for each hypothesis and the *P* values of the tests are reported in the Table A2.

*Bicyclus anynana* displayed significant genetic variation between populations, yet smaller when using the mitochondrial COI marker than the CAD nuclear marker. The ML phylogenetic reconstructions showed that the genetic divergence for COI was not associated to the geographic origin of the populations (Fig. A2) and constraining the COI tree to display geographic clades significantly decreased its likelihood (S.H. test: *P *<* *0.01; Table A2). In contrast, the ML phylogenetic tree using the CAD gene separated most of the Ugandan haplotypes from other localities (BT of 80% for the Ugandan clade). Preventing Ugandan haplotypes to form a clade in the CAD tree significantly decreased its likelihood (S.H. test: *P *=* *0.03; Table A2).

The ML phylogenetic reconstructions using COI supported the genetic differentiation of geographic clades in *B. smithi* (bootstrap support, BT hereafter, of 86% for separating Uganda from Cameroon) and *B. vulgaris* (BT of 71% for grouping Nigerian haplotypes, Fig. A2). The geographically intermediate *B. vulgaris* from Cameroon grouped with either Nigerian or Ugandan haplotypes showing the coexistence of the two mitochondrial lineages in Cameroon. For the two species, the clade separation in the COI gene was supported by S.H. tests (Table A2). In contrast, the CAD gene showed no genetic differentiation between populations of the two species. *B. safitza* did not display geographic clade either for COI or for CAD, despite the distance separating Nigeria and Uganda (approximately 2600 km, Figs. A1, A2). Constraining the tree for the presence of the clades did not improve the likelihood, and SH test revealed no significant differences (Table A2).

**Table A2:** Likelihood scores (log *L*) of ML trees with and without positive or negative constraint

Species	COI gene				CAD gene			
	Constraint	Constrained groups	log *L*	*P*	Constraint	Constrained groups	log *L*	*P*
*Bicyclus anynana*	None	None	−1873.6		None	None	−1565.2	
	Positive	(Ug) (Ke + SA)	−1881.1	<0.01	Negative	(Ug) (Ke + SA)	−1574.7	0.03
*Bicyclus safitza*	None	None	−1748.5		None	None	−1378.3	
	Positive	(Ni) (Ug)	−1751.7	0.24	Positive	(Ni) (Ug)	−1382.9	0.30
*Bicyclus smithi*	None	None	−2139.5		None	None	−1489.6	
	Negative	(Ca) (Ug)	−2160.9	<0.01	Positive	(Ca) (Ug)	−1536.4	<0.01
*Bicyclus vulgaris*	None	None	−1644.1		None	None	−1402.2	
	Negative	(Ni) (Ug)	−1669.3	<0.01	Positive	(Ni) (Ug)	−1407.7	0.20

Constrained groups of populations are delimited by parentheses. The *P* value of the S.H. test to compare the trees is reported in the last column for each gene. For the CAD in *B. anynana*, three haplotypes are omitted in the constraint of the Ugandan individuals. These three CAD haplotypes are present in Ugandan individuals together with typical Ugandan haplotypes but more similar to Kenyan and South African ones (pointed by black arrows in Figs. 2 and A2). These three haplotypes are likely introgressed from the southern populations and the three individuals are likely hybrids (see Results in Main Text and Appendix 5).

## Appendix 3 Congruence between COI and the nuclear markers in *B. anynana*

To assess whether the COI or the CAD marker was a better signal of the evolutionary history of *B. anynana*, we obtained additional sequences for the nuclear marker EF1*α* for seven Ugandan individuals, two South African individuals from False Bay, and three from Limpopo (Tables 1 and S1). We measured the average number of pairwise differences between all individuals for each of the three genes. The average divergence between populations is larger than the average divergence within populations for the two nuclear markers, but not for the COI (Table A3). This confirmed the deep nuclear divergence found for Ugandan individuals compared to more southern populations, which is also in accordance with the description of a Ugandan subspecies, using morphological traits, *B. anynana centralis*, Condamin, 1968. As COI is supposed to evolve faster than the nuclear markers (EF1*α* is used to resolve deep nodes in phylogeny studies in butterflies), this results mean that COI is unreliable to describe the neutral genetic divergence of the populations in this species. In the three other species investigated, COI displays, as expected, a larger divergence between populations (Appendices 2 and 4).

**Table A3:** Number of pairwise differences between individuals for the three genes

Pairwise differences	COI	CAD	EF1*α*
Within Uganda	0.86 ± 0.48	0.19 ± 0.13	0.00 ± 0.00
Within South Africa	1.40 ± 0.82	2.10 ± 0.83	0.30 ± 0.28
Between Uganda and South Africa	3.23 ± 1.48	6.49 ± 2.16	2.23 ± 0.90
Ratio between/within	1.43	2.83	7.43

This analysis was performed with MEGA5 and the standard error computed with 1000 bootstrap (Tamura et al. 2011). The ratio between the number of differences between and within population is stronger in CAD than in COI in accordance with the ANOVA and pairwise Φ_ST_ results.

This relative mitochondrial uniformity in *B. anynana* suggests a recent spread of one haplotype across populations (mitochondrial introgression). Such a pattern of mitochondrial discordance is not uncommon and justifies the use of several independent markers (Funk and Omland 2003; Bazin et al. 2006). The erasing of mitochondrial diversity can be due to drift (stronger in the mitochondrial genome with lower effective population size due to haploidy and maternal inheritance), selection of an advantageous mutation (selective sweep facilitated by the absence of recombination in this genome), or selection by symbiotic interaction with bacteria such as *Wolbachia* (reviewed in Ballard and Whitlock 2004). According to de Jong et al. (2011), the neutrality of the polymorphism in COI sequences was not met for several *B. anynana* populations, which would reject the first hypothesis of fixation by drift. We applied neutrality tests *H* of Fay and Wu (2000) and *D* of Tajima (1989) with the DnaSP software (version 5.10.01; Librado and Rozas 2009) and observed that both were significant for the COI but not for the CAD gene (Table A4). This further supported the hypothesis of a recent sweep on the mitochondrial polymorphism.

**Table A4:** Neutrality tests

Gene	Tajima (1989)		Fay and Wu (2000)	
	*D*	*P*	*H*	*P*
CAD	−1.46	>0.10	0.61	0.40
COI	−2.39	<0.01	−16.81	<0.01

*P* values of the *H* test were computed from 1000 coalescent simulations based on the observed diversity (theta) value. Both tests were performed with DnaSP.

## Appendix 4 Genetic differentiation in the four *Bicyclus* species

The median joining networks confirmed the genetic structure of the three species as found in the trees: We found a neat COI geographic differentiation in *B. smithi* and *B. vulgaris*, while there was no pattern of genetic structure for *B. safitza* despite the large geographic area sampled (Fig. A2). *Bicyclus safitza* is abundant and well adapted to anthropogenic ecosystems (observations in Makerere University campus in Kampala, Uganda, and see also Munyuli 2012), such that it may experience little geographic or ecological barrier to gene flow despite its large geographic distribution.

The AMOVA and pairwise Φ_ST_ between populations supported in general the COI genetic differentiation observed in trees and networks between populations in *B. smithi* and *B. vulgaris*. In *B. smithi*, AMOVA and individual Φ_ST_ additionally revealed a significant divergence for the CAD (Table A5). Despite no separation into clades, in *B. safitza*, the AMOVA and pairwise Φ_ST_ detected a significant effect of geography in the distribution of genetic diversity (46% and 21% of the variance explained for COI and CAD, respectively, Table A5).

Of note, the divergence between the mitochondrial clades found within *B. smithi* and *B. vulgaris* (average uncorrected pairwise difference of 1.96 ± 0.34% SD and 1.53 ± 0.17%, respectively) was under the level known for other Lepidopteran subspecies (3.4% in *Heliconius erato*, Brower, 1994; or 2% in *Spodoptera frugiperda* host plant strains, Kergoat et al., 2012), suggesting that we are dealing with intraspecific genetic variability.

**Table A5:** Summary of the genetic variation between pairs of populations of the four species for the COI and CAD genes

Species	Pairs of populations		COI			CAD		
			Clade support	AMOVA	Φ_ST_	Clade support	AMOVA	Φ_ST_
*Bicyclus anynana*	Uganda	Kenya	Positive	Among: **20%** *******	**0.43** *******	Negative	Among: **37%** *******	**0.56** *******
	Uganda	South Africa False Bay	***P*** ** < 0.01**	Within: 80%	**0.09** *******	***P*** ** = 0.03**	Within: 4%*****	**0.54** *******
	Uganda	South Africa Limpopo	No Ugandan clade		**0.04** *******	Ugandan clade	Within ind.: **59%** ******	**0.57** *******
	Kenya	South Africa False Bay			**0.30** *******			0.00
	Kenya	South Africa Limpopo			**0.28** *******			0.00
	South Africa False Bay	South Africa Limpopo			**0.04** ******			0.00
*Bicyclus smithi*	Cameroon	Uganda	Negative	Among: **66%** *******	**0.66** *******	Positive	Among: **9%** *******	**0.10** ******
			***P *** **<** *** *** **0.01**	Within: 34%		***P *** **<** *** *** **0.01**	Within: 5%	
			Two clades			No clade	Within ind.: 85%	
*Bicyclus vulgaris*	Nigeria	Uganda	Negative	Among: **94%** *******	**0.94** *******	Positive	Among: 5%	
			***P *** **<** *** *** **0.01**	Within:6%		*P *=* *0.20	Within: **24%** *******	
			Two clades			No clade	Within ind.: **72%** *******	
*Bicyclus safitza*	Nigeria	Uganda	Positive	Among: **46%** *******	**0.46** *******	Positive	Among: **21%** *****	
			*P *=* *0.24	Within: 54%		*P *=* *0.30	Within: **30%** *******	
			No clade			No clade	Within ind.: **49%** *******	

The number of SNPs for COI and CAD genes is 34 and 51 in *B. anynana*, 27 and 31 in *B. safitza*, 60 and 33 in *B. smithi*, and 39 and 42 in *B. vulgaris*. Clade supports are based on SH test, and we reported the sign of the constraint, the significance of the decrease of the likelihood, and the supported conclusion (detailed method and results in Appendix 2). AMOVA and pairwise Φ_ST_ are based on uncorrected pairwise genetic differences. For AMOVA, the displayed values correspond to percentage of variation among populations, within populations, and in addition for CAD, within individuals. Significant values are in bold (asterisks inform on significance of the estimates: “*” if *P *≤* *0.05; “**” if *P *≤* *0.01; “***” if *P *≤* *0.001).

## Appendix 5 Ugandan heterozygotes using the CAD gene of *B. anynana*

The three CAD haplotypes sampled in Uganda but present out of the Ugandan clade were associated to typical Ugandan haplotypes in three heterozygous individuals. These three Ugandan individuals had heterozygous positions for two and three of the three positions that bore, in all other sampled *B. anynana* individuals, fixed differences between the Ugandan and the Kenyan and South African populations (Table A6). The three heterozygous individuals also presented a heterozygous state for two other positions fixed in Ugandan individuals but still variable in more southern populations (Table A6). Given the frequency of these mutations, the probability of being heterozygous (by mutation) for at least three of these five positions was very low in the Ugandan population (*P *=* *0.008, 0.0004, and 8.56 × 10^−6^ for three, four, and five heterozygous positions as observed for these three individuals, Table A7). It was even more surprising to observe no individual heterozygous for only one or two positions (*χ* ^2^ = 5128.61, *P *=* *0.0001 obtained from 10,000 Monte Carlo simulations; Table A8). Thus, the probability that these three individuals bear alleles both typical from Uganda and from other populations, which differ at several positions, due to mutation only is very low. It is far more likely that they inherited these heterozygous genotypes from recent hybrid matings between Ugandan and more southern individuals. Based on the strong divergence of the clades at the nuclear level, the differentiation of the populations must be ancient compared to pairs of populations investigated in the other species (Appendix 4), yet reproductive isolation is not yet complete given the presence of the three hybrids.

**Table A6:** Number of individuals displaying the different genotypes in Uganda and in the other populations (South Africa/Kenya) for five nucleotide positions in the CAD gene

Clade	Position 105		Position 212		Position 341		Position 461		Position 677	
Uganda *n* = 23	CC	20	TT	20	CC	20	AA	20	AA	20
Ind. Mu005	CC	1	TT	1	AC	1	CA	1	TA	1
Ind. Iu043	AC	1	AT	1	AC	1	CA	1	AA	1
Ind. Iu049	AC	1	AT	1	AC	1	CA	1	TA	1
Local proportion of state	C	0.96	T	0.96	C	0.93	A	0.93	A	0.96
South Africa/Kenya *n* = 52	CC	0	TT	9	CC	0	AA	18	AA	0
	CA	4	TA	28	CA	0	AC	20	AT	0
	AA	48	AA	15	AA	52	CC	13	TT	52
							TC	1		
Local proportion of state	A	0.96	A	0.56	A	1.00	C	0.45	T	1.00

The nucleotide positions correspond to the 714 base pairs considered for the CAD gene. In Uganda, the character states are fixed for the five positions except for three individuals (“Mu005”, “Iu043”, and “Iu049”, arrows in Figs. 2 and A2). These five mutations are synonymous.

**Table A7:** Probability of observing different combinations of genotypes at the five positions of CAD fixed in Uganda

Number of heterozygous positions	Probability of observation in Uganda	Expected	Observed
0/5	0.58	14	20
1/5	0.32	8	0
2/5	0.07	2	0
3/5	0.0076	0	1
4/5	0.0004	0	1
5/5	0.000009	0	1

For example, the probability of being fully homozygous for the more frequent state at these five positions is the product of the squared probability of the states at each position (that is: *P*(*C*_pos.105_)^2^ × *P*(*T*_pos.212_)^2^ × *P*(*C*_pos.341_)^2^ × *P*(*A*_pos.461_)^2^ × *P*(*A*_pos.677_)^2^; or: 0.96^2^ × 0.96^2^ × 0.93^2^ × 0.93^2^ × 0.96^2^ = 0.58).

**Table A8:** Chi‐squared tests’ designs and results

Contingency table subset	*χ²*	*P* value estimation method	Degrees of freedom	*P* value
Full table	5128.61	10,000 Monte Carlo simulations	NA	9.9 × 10^−5^
1 or no heterozygous position	11.04	Conventional	1	0.0009
1–5 heterozygous positions	15,973.58	10,000 Monte Carlo simulations	NA	9.9 × 10^−5^

When chi‐squared test's *P* value could not be obtained by conventional method, Monte Carlo simulations were used.

## Appendix 6 Chemical differentiation in the the four *Bicyclus* species studied

The full chemical profile of *B. safitza* and *B. vulgaris* displayed a similar number of compounds to *B. anynana* (i.e., from 8 to 16 in males and from 1 to 6 in females), while we found a larger number of compounds in *B. smithi* (18.8 ± 3.4 and 9.8 ± 5.4 for males and females, respectively; Table A9).

In these three species, the repeatability of the male chemical profiles within populations ranged between 0.49 ± 0.24 and 0.70 ± 0.15 (mean ±* *SD, Spearman correlations, *n *=* *3 males per population) except for *B. safitza* and *B. vulgaris* from the Nigerian populations (0.19 ± 0.30 and 0.37 ± 0.23, respectively).

One to five pMSP components were selected in the different *B. safitza*,* B. smithi*, and *B. vulgaris* populations (Fig. A1). Similarly to *B. anynana*,* B. vulgaris* displayed differences in pMSP composition between populations: pMSP12 and pMSP14 were absent from *B. vulgaris* in Nigeria (Fig. A1; Table A2). In *B. safitza* and *B. smithi*, the observed differences in pMSP composition between populations were not due to the absence of some compounds but to variations in their amounts that determined whether they were selected as pMSP components or not. Because of the small sample size, PerMANOVA analyses were not informative for *B. safitza*,* B. smithi*, and *B. vulgaris* (Table A10). Yet, in *B. vulgaris*, 84% (PerMANOVA, *n *=* *6, *P *=* *0.10) of the qualitative variation in pMSP composition among individuals was explained by geography, and this was based on the absence of the two compounds in the Nigerian population. *B. safitza* seemed to have strong differences in pMSP absolute amounts with 48% explained by geography (PerMANOVA, *n *=* *6, *P *=* *0.10).

In conclusion of the preliminary analysis of these three species, the pattern of qualitative chemical differentiation observed between populations of *B. anynana* was not anecdotal as present in two of four species. It has to be kept in mind that given the small sample size in *B. vulgaris*, we cannot exclude that part of the qualitative variation in pMSP composition between the two populations was due to chance.

**Table A9:** Summary of the number of compounds per population and sex

Number of compounds	*Bicyclus anynana*											
	Males						Females					
	Um	K	M	SAl	SAf	SAflab	Um	Ke	Ma	SAl	SAf	SAflab
Per individual												
Maximum	11	12	14	18	15	19	4	5	6	4	4	2
Mean	9.0	9.6	12.3	16.0	12.4	14.7	3.5	2.0	5.5	2.5	2.5	2.0
Minimum	6	7	9	13	9	9	3	1	5	2	1	2
Mean ± SD of the sex	12.1 ± 3.2						2.6 ± 1.2					
Per location	14	22	15	29	25	20	4	5	6	4	6	2
Mean ± SD of the sex	20.8 ± 5.8						4.5 ± 1.5					

Number of compounds	*Bicyclus safitza*				*Bicyclus smithi*				*Bicyclus vulgaris*			
	Males		Females		Males		Females		Males		Females	
	N	Um	N	Um	Cw	Uk	Cw	Uk	N	Um	N	Um
Per individual												
Maximum	12	19	4	8	21	21	11	16	10	23	2	9
Mean	9.3	15.0	3.5	6.0	17.3	20.3	7.7	13.0	7.3	21.0	1.5	7.5
Minimum	7	12	3	4	12	20	1	10	5	19	1	6
Mean ± SD of the sex	12.2 ± 4.2		4.8 ± 2.2		18.8 ± 3.4		9.8 ± 5.4		14.2 ± 7.8		4.5 ± 3.7	
Per location	19	21	5	9	26	31	13	17	15	30	2	9
Mean ± SD of the sex	20.0 ± 1.4		7.0 ± 2.8		28.5 ± 3.5		15.0 ± 2.8		22.5 ± 10.6		5.5 ± 4.9	

Compounds were counted after the removal of small peaks <10% IS. Populations are coded as follows: Um and Uk for Mburo and Kibale in Uganda; K for Kenya; M for Malawi; SAf, SAflab and SAl for False Bay (wild caught and laboratory reared) and Limpopo in South Africa; N for Nigeria, Cw for western Cameroon.

**Table A10:** Distribution of the chemical variation of male pMSP (above) and female chemical profiles (below) explained by the geographic origin of populations in *Bicyclus safitza*,* Bicyclus smithi*, and *Bicyclus vulgaris*

Species	Sex	Compounds	Factors	Presence–absence	Absolute abundance	Relative abundance
				*R* ^2^ (%)	*R* ^2^ (%)	*R* ^2^ (%)
*B. safitza*	Males	Full profile	Location	42	43	8
		pMSP	Location	20	48	6
	Females	Full profile	Location	50	85	90
*B. smithi*	Males	Full profile	Location	51	50	60
		pMSP	Location	20	23	25
	Females	Full profile	Location	44	35	24
*B. vulgaris*	Males	Full profile	Location	46	36	31
		pMSP	Location	84	36	35
	Females	Full profile	Location	79	81	38

PerMANOVA analyses were performed for each type of chemical distance (presence–absence, absolute, and relative abundances, respectively). None of these results were significant.
