# Supplementary material for: Differentiation in putative male sex pheromone components across and within populations of the African butterfly Bicyclus anynana as a potential driver of reproductive isolation
Source: Ecol Evol. 2016 Jul 29;6(17):6064–84. doi: 10.1002/ece3.2298 (PMC5016632; doi:10.1002/ece3.2298)

Figure S3: Mass spectra of the pMSP components.

The second mass spectra below each pMSP component (except MSP3 to pMSP6, pMSP9, pMSP12 and pMSP16) belong to reference compounds for comparison sake.

MSP1 – *B. anynana*:

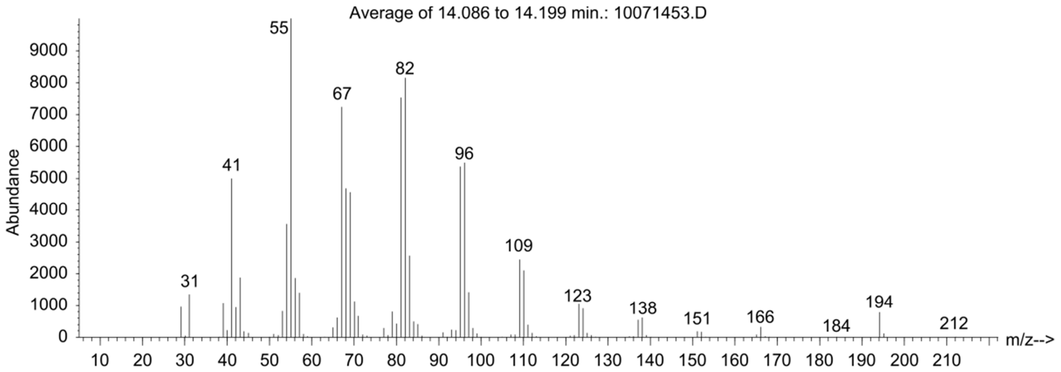

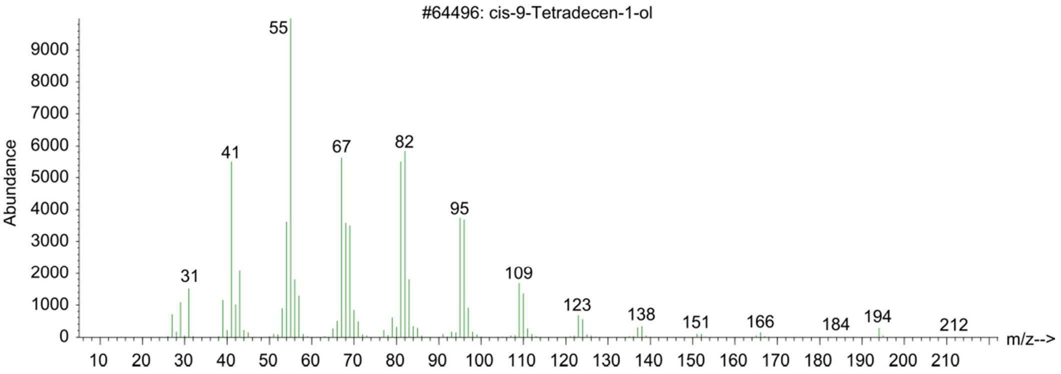

MSP2 – *B. anynana*:

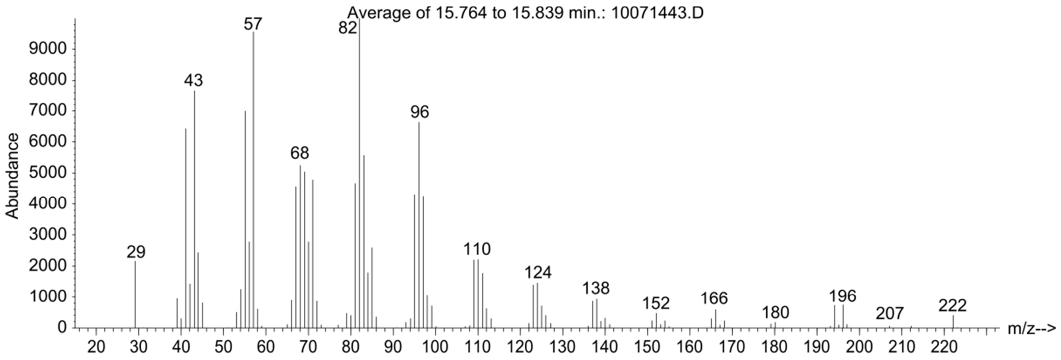

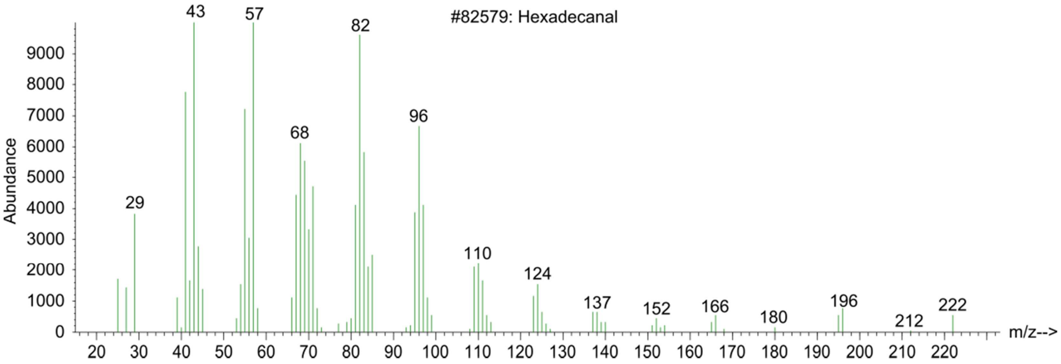

MSP3 – *B. anynana*:

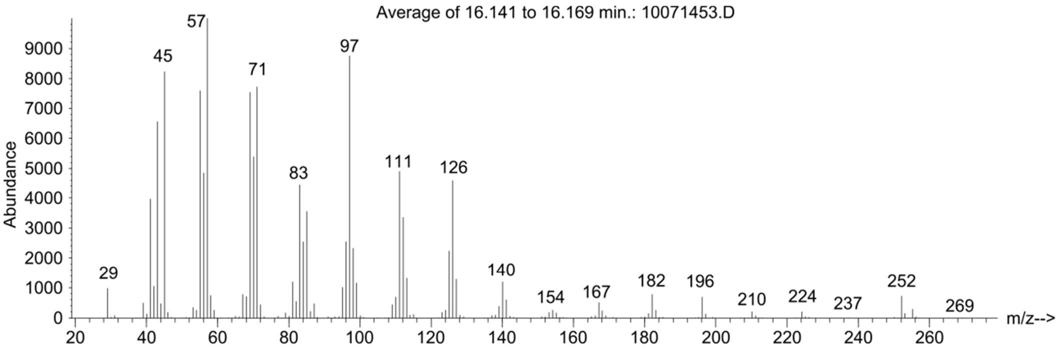

pMSP4 – *B. anynana*:

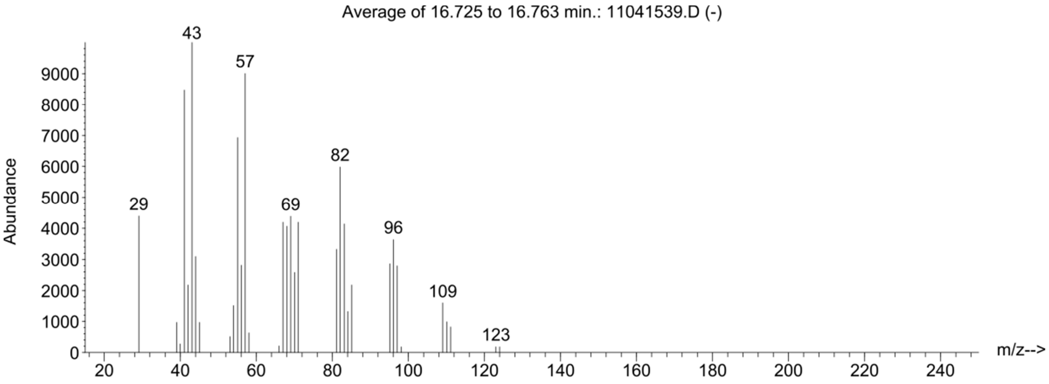

pMSP5 – *B. anynana*:

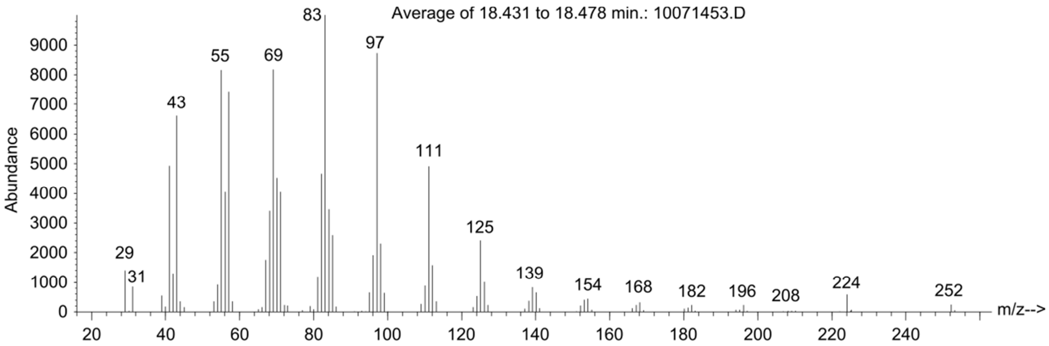

pMSP6 – *B. safitza*:

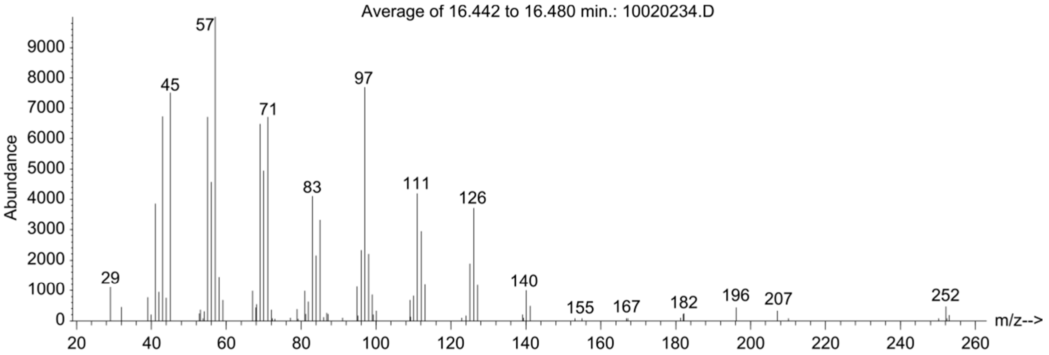

pMSP7 – *B. safitza*:

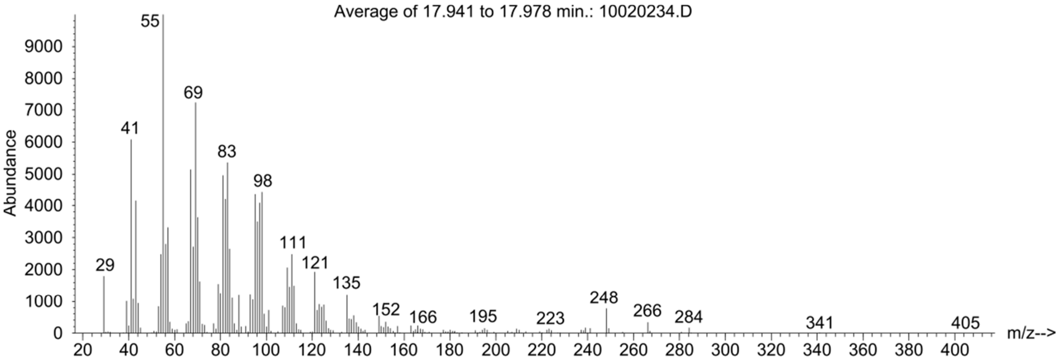

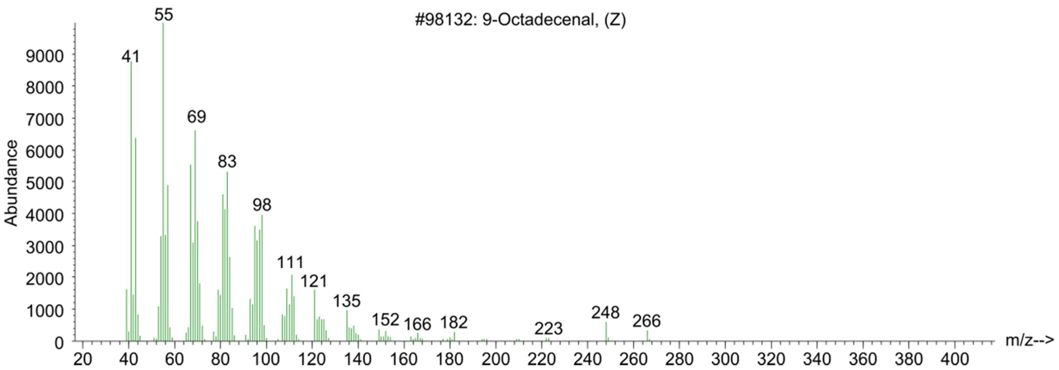

pMSP8 – *B. safitza*:

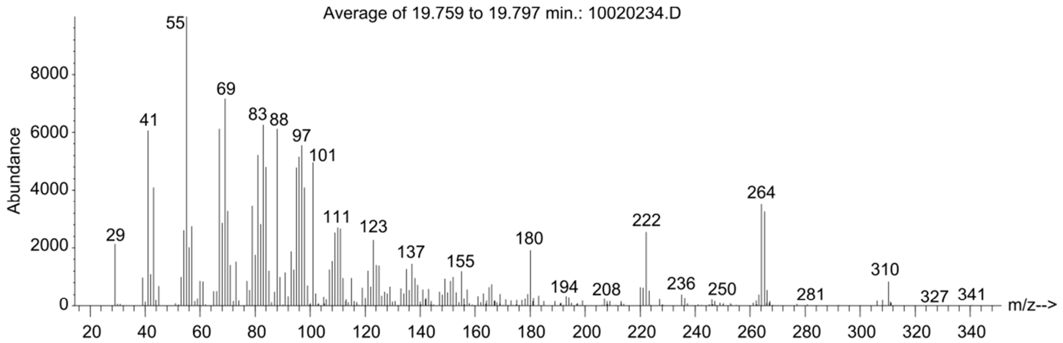

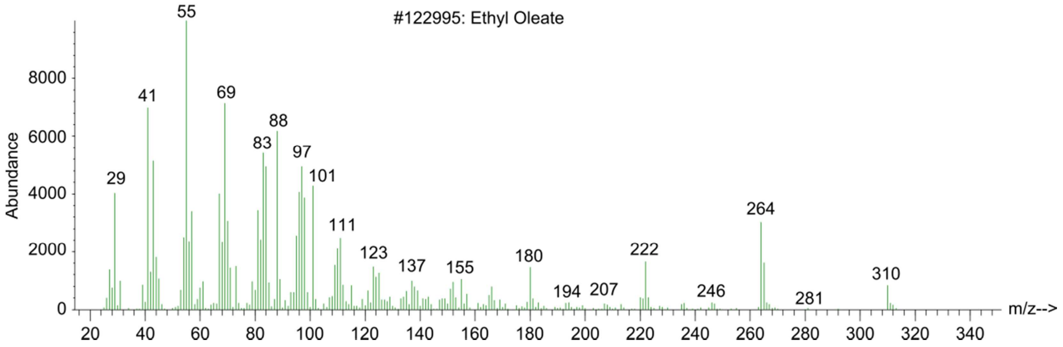

pMSP9 – *B. smithi*:

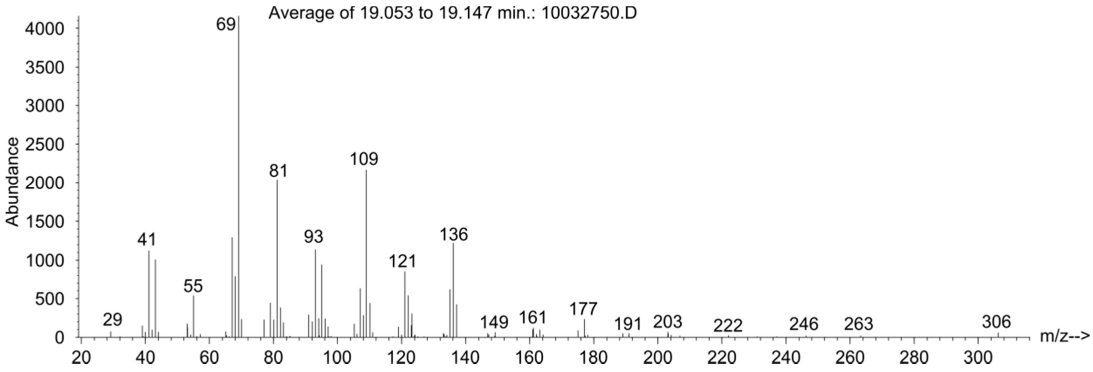

pMSP10 – *B. smithi*:

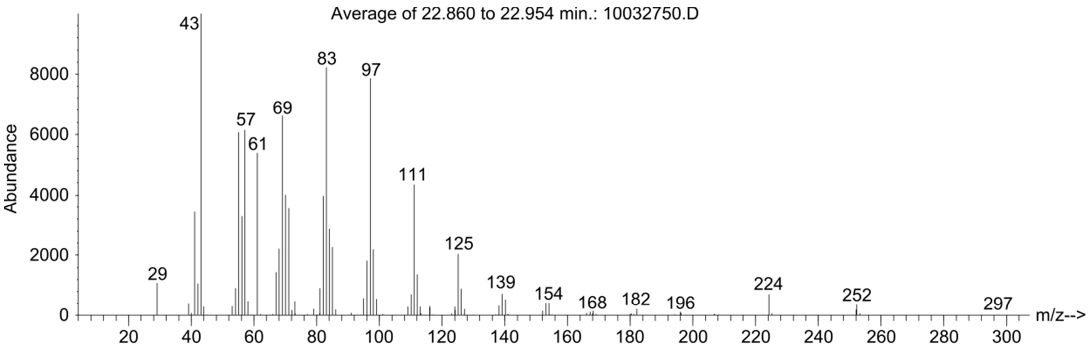

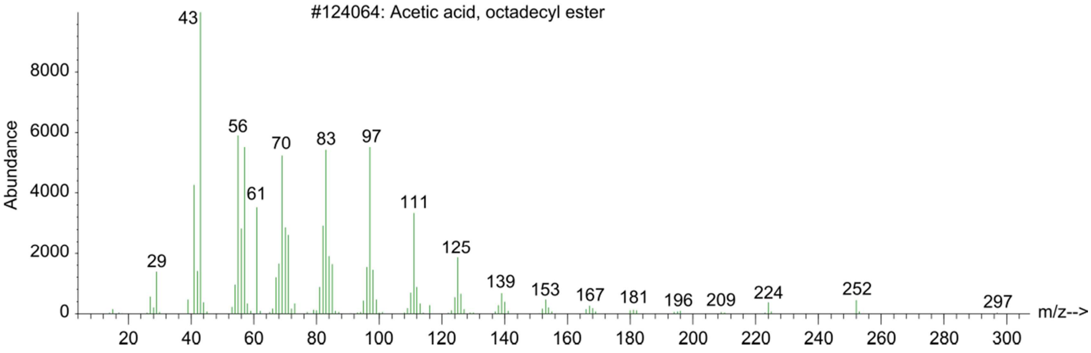

pMSP11 – *B. smithi*:

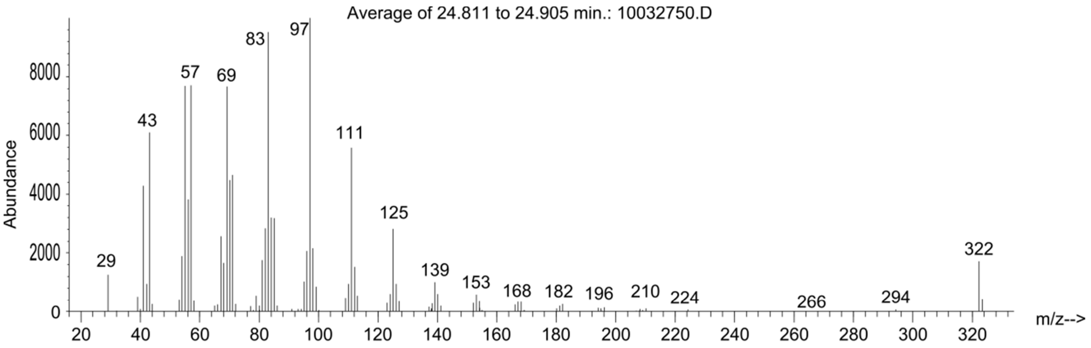

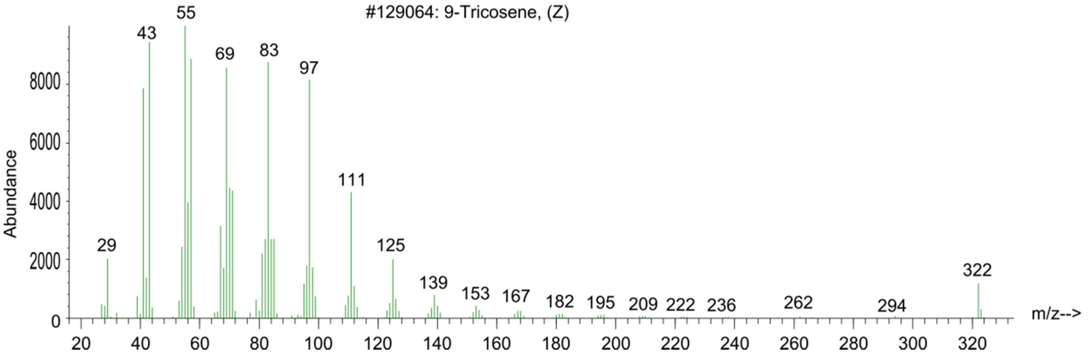

pMSP12 – *B. vulgaris*:

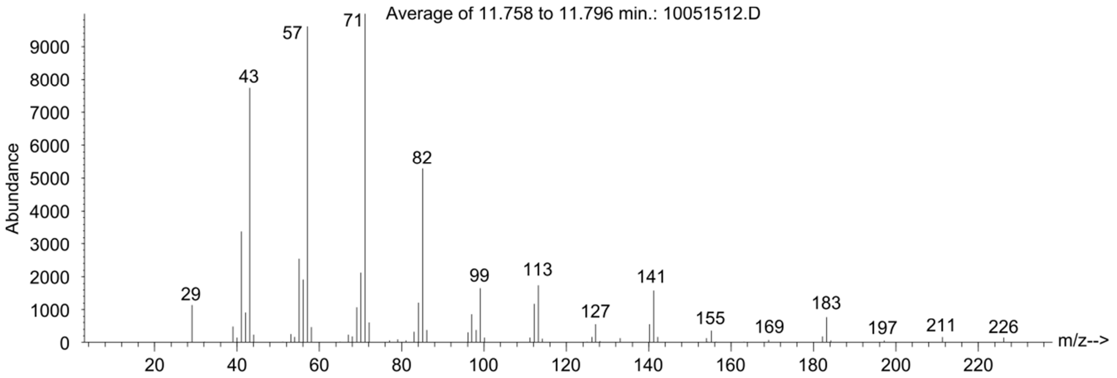

pMSP13 – *B. vulgaris*:

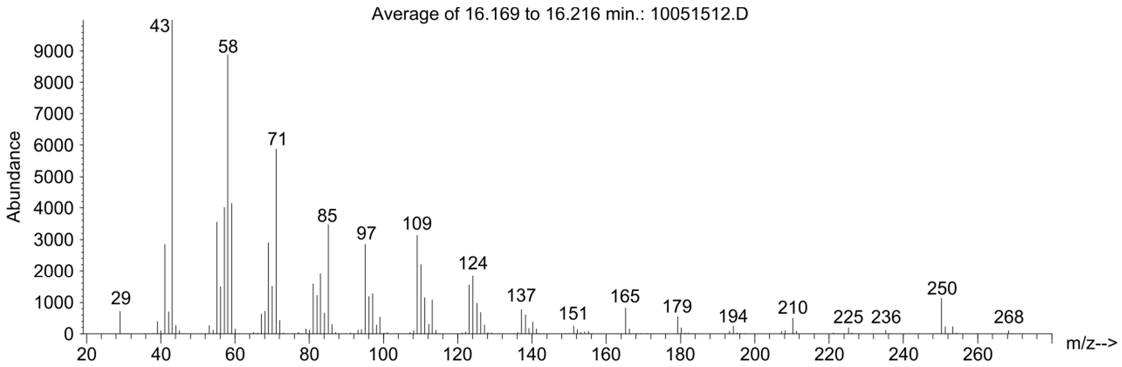

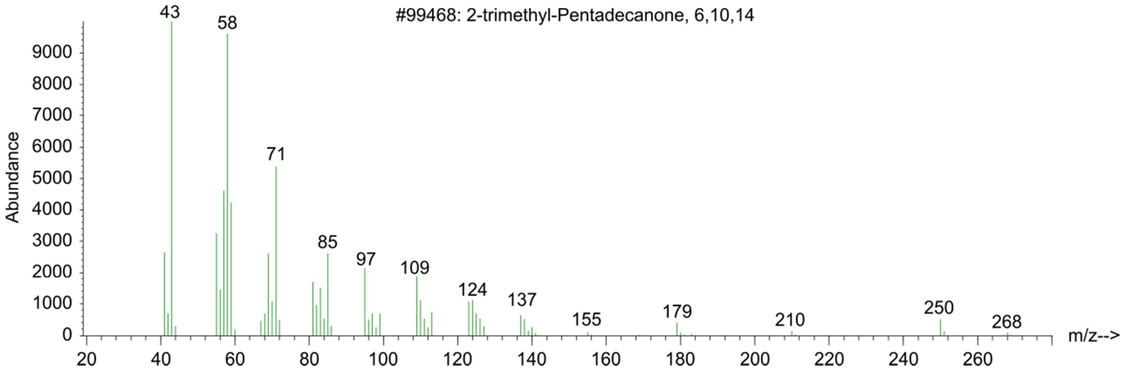

pMSP14 – *B. vulgaris*:

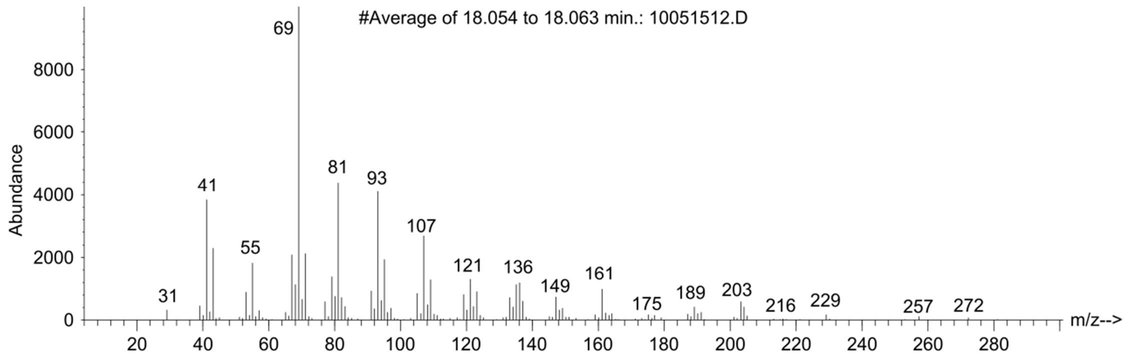

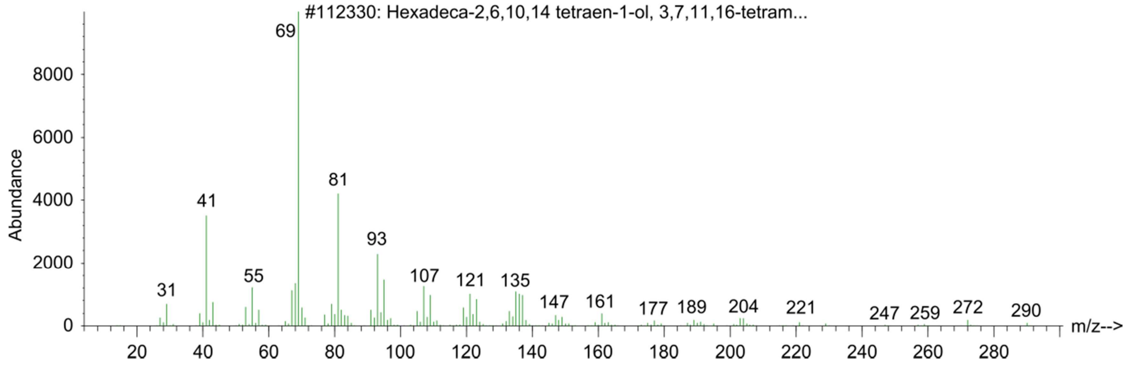

pMSP15 – *B. vulgaris*:

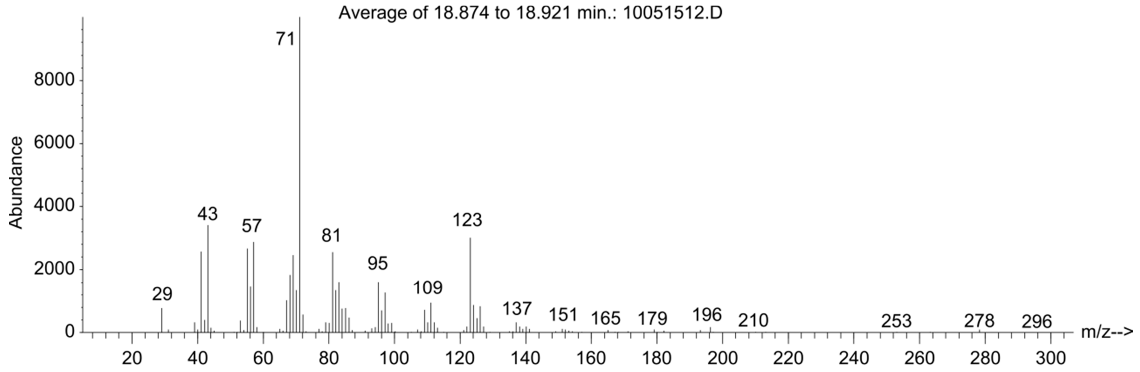

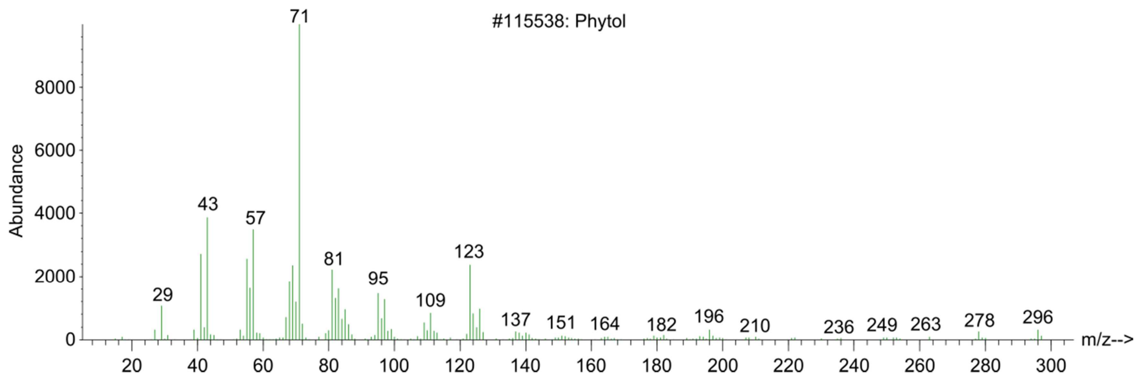

pMSP16 – *B. vulgaris*:

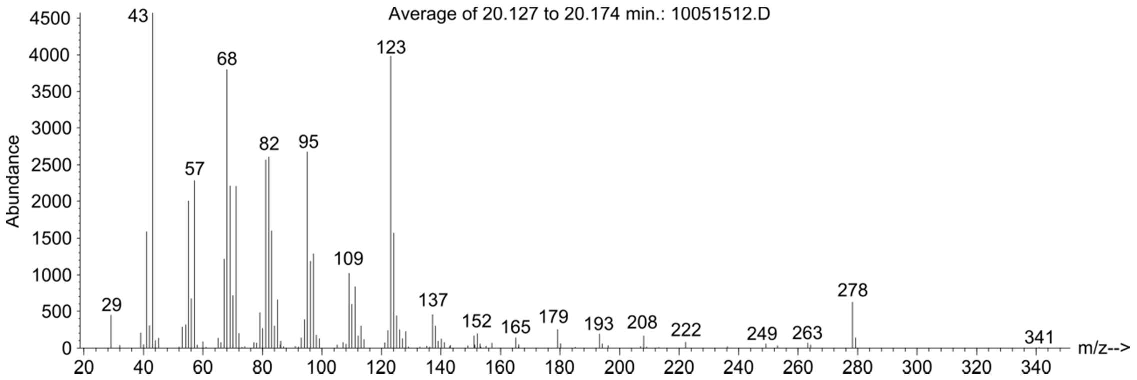

Supplement: Supplementary file 1 — Figure S1. Mass spectra of the pMSP components. [file ECE3-6-6064-s001.pdf]
