# Supplementary material for: Differentiation in putative male sex pheromone components across and within populations of the African butterfly Bicyclus anynana as a potential driver of reproductive isolation
Source: Ecol Evol. 2016 Jul 29;6(17):6064–84. doi: 10.1002/ece3.2298 (PMC5016632; doi:10.1002/ece3.2298)
Supplement: Supplementary file 4 [file ECE3-6-6064-s004.docx]

**Legend to supplementary Information files**

**Supplementary Table 1:** Detailed list of sampled individuals for the four *Bicyclus* species.

For each individual, we listed the precise geographic origin (country, location, GPS coordinates), the date of collection, the collector, the sequences obtained (gene and reference of the primers) with related GenBank accession numbers, the haplotype code corresponding to the trees (Appendix Figure 2), and correspondence with chemical sampling (‘old GC-MS’ refers to Ugandan additional *B. anynana* males mentioned in the main text). The seven individuals that showed variable results across different runs of the ELB phasing algorithm on CAD sequences are mentioned in the table (two of *B. anynana*, *B. smithi* and *B. vulgaris*, and one *B. safitza*). These individuals originated either from the additional populations not sampled for chemical profiles (Cameroon and Ishasha, Uganda) or, in the case of *B. anynana*, from the False Bay population in South-Africa, one of the most numerously sampled of our study (37 individuals). We are thus convinced that it does not impact our results.

**SupplementaryTable 2**: List of compounds detected in the four species.

This table lists all compounds present above the threshold of 10% of the internal standard and their abundance in each individual of the four species. The pMSP components are highlighted in the first column. The subsequent columns describe the type of the compounds (chemical family), their assigned structure and the details of their identification (type of column for the GC, representative mass fragments). All GC-MS files are available upon request.

**Supplementary Figure 1**: Mass spectra of the pMSP components.

The second mass spectra below each pMSP component (except MSP3 to pMSP6, pMSP9, pMSP12 and pMSP16) belong to reference compounds for sake of comparison.
